# Automated Design of Complex Dynamic Systems

**DOI:** 10.1371/journal.pone.0086696

**Published:** 2014-01-31

**Authors:** Michiel Hermans, Benjamin Schrauwen, Peter Bienstman, Joni Dambre

**Affiliations:** 1 ELIS department, Ghent University, Ghent, Belgium; 2 INTEC department, Ghent University, Ghent, Belgium; Universidad de Zarazoga, Spain

## Abstract

Several fields of study are concerned with uniting the concept of computation with that of the design of physical systems. For example, a recent trend in robotics is to design robots in such a way that they require a minimal control effort. Another example is found in the domain of photonics, where recent efforts try to benefit directly from the complex nonlinear dynamics to achieve more efficient signal processing. The underlying goal of these and similar research efforts is to internalize a large part of the necessary computations within the physical system itself by exploiting its inherent non-linear dynamics. This, however, often requires the optimization of large numbers of system parameters, related to both the system's structure as well as its material properties. In addition, many of these parameters are subject to fabrication variability or to variations through time. In this paper we apply a machine learning algorithm to optimize physical dynamic systems. We show that such algorithms, which are normally applied on abstract computational entities, can be extended to the field of differential equations and used to optimize an associated set of parameters which determine their behavior. We show that machine learning training methodologies are highly useful in designing robust systems, and we provide a set of both simple and complex examples using models of physical dynamical systems. Interestingly, the derived optimization method is intimately related to *direct collocation* a method known in the field of optimal control. Our work suggests that the application domains of both machine learning and optimal control have a largely unexplored overlapping area which envelopes a novel design methodology of smart and highly complex physical systems.

## Introduction

The digital computation paradigm has become so dominant, that in the minds of many, the word digital is implicitly assumed whenever computation is mentioned. This is mainly due to the fact that digital computation is extremely robust against variability and noise. This greatly facilitates the design process and is one of the main reasons why we can keep on designing ever more complex computers. However, the digital paradigm doesn't map well to the natural computation that occurs in many physical media and the quenching of their often rich dynamical spectrum to two-valued attractor dynamics comes at a huge efficiency cost. In contrast, analogue computers carry the potential to directly exploit the way the dynamics of physical systems respond to external stimuli, continuously transforming their real-valued state. This requires the selection of a physical system with natural dynamics that roughly match the computational requirements of a given task. Some of the pioneers of current computer science have investigated more generally applicable analogue computing models. For instance, in Von Neumann's original discussion of cellular automata [1], five types were proposed, most of which were analogue. Turing's description of the role of reaction-diffusion in morphogenesis [2] is another example. This work was originally adopted mainly by the biological community (to study morphogenesis), but it later became the basis for, e.g., Adamatzki's recent work on reaction-diffusion computers [3].

In practice, even coming close to the exploitation of this potential for complex behaviors that are not easily partitioned into small building blocks requires an economically unacceptable design effort. Besides the inherent complexity of tuning a complex nonlinear dynamical system, designers need to ensure robustness under uncertain conditions. Many design parameters are not very well controlled during fabrication. Additionally, they may vary in time in random or systematic ways (e.g., due to thermal effects). Finally, the exact desired behavior of the system can usually not be described analytically, because it needs to perform its task under variable conditions and in the presence of what is usually termed *noise*.

Yet, robust and highly complex analogue computing occurs within all living systems, from single cells to complex organisms. As the brain (human or animal) is exposed to stimuli from its senses, muscles and pain receptors, it auto-rewires, using its adaptation mechanisms, first in order to structure, correlate and organize the vast amounts of incoming data and then to control its actions in order to achieve increasingly complex goals. Less well known is the fact that animal bodies are constructed in such a way that their movements require as little energy as possible and can mostly be controlled by relatively simple periodic central pattern generators (CPGs - [4], [5]). This is being exploited in a recent trend in robotics called morphological computing [6]–[8], in which robot designers focus on the design of robots rather than their control. The central claim of this line of research is that a large part of the control complexity can be internalized into the robot's morphology by clever design.

Many efforts to solve such problems have been made in the past. One often used design strategy, inspired by the evolution that led to biological systems, is to use metaheuristic optimization. This term refers to algorithms that treat the system as a black box, only sample the solution space and use some heuristic search algorithm to maximize an associated fitness function, e.g., evolutionary techniques. One issue with this approach is that truly large-scale systems with thousands of parameters offer a too large search space, and the time needed for optimizing grows prohibitively large.

In this paper we introduce a design methodology that allows for a more efficient design of robust physical systems. Our approach is applicable whenever an approximate parametric model of the system's dynamics exists and sufficient examples of the desired dynamical behavior are available. Essentially, we revert to machine learning algorithms, which have proven their merit in creating remarkably powerful systems, and apply them to physical dynamic systems, operating in continuous time. In particular we extend the gradient descent training algorithms known as *Real-Time Recurrent Learning* (RTRL), and *Backpropagation through time* (BPTT), respectively. These are commonly used for training recurrent neural networks (RNNs), which are discrete-time dynamical systems. Historically, BPTT was introduced first [9]–[12], and it was developed by eliminating the time-aspect of recurrent neural networks and considering them a special form of multi-layered perceptron. RTRL was introduced later [13] as an online alternative to BPTT. BPTT has proven to be a highly successful method for training recurrent networks, leading to remarkably complex and powerful systems [14], [15], often with several millions of parameters. As we show, our extensions of BPTT and RTRL are capable of taking into account and exploiting the long-term dynamic effects of the systems under consideration.

The resulting equations that describe how the gradients w.r.t. the parameter values are computed are identical to equations that are used within numerical optimal control, more specifically the computation of the costates of the system, which stem from the Pontyagrin maximum principle [16].

Optimal control primarily deals with a different problem: given a certain dynamic system, how can we create an input (or control) signal for this system in order to get it to operate in a certain way. This problem has a wide variety of applications in chemical plants, economy, robotics, spaceflight, etc. one example is the minimization of fuel expenditure in a rocket leaving the earth's atmosphere [17]. Even though the mathematical formalism also allows for the optimization of system parameters, not much work actually considers optimal control as a useful tool for system design, and much more commonly systems are designed first, and later optimal control algorithms are applied to control them. Machine learning starts from an information processing perspective. Here, the input signals of the system need to be processed (filtered, classified,…). Samples of desired input-output behavior are provided, and the *system parameters* (usually static values) need to be optimized in order to optimally approximate this desired behavior.

The design problems presented in this paper lie within the overlapping area of machine learning and optimal control: optimizing the *design* rather than control, but working with physical dynamical systems. We provide a set of three examples of which one leans to control theory (locomotion), one to optimal design (magnetic beam focusing), and one to machine learning (optimizing a photonic chip using examples of input/output signals).

## Results

In this section, we first provide the equations needed for obtaining gradients in continuous time dynamical systems, and we explain how they can be used in an online and offline optimization fashion. Second, we illustrate the applicability of our approach by optimizing three different dynamical systems.

### Gradient Descent

Here we briefly present the main mathematical results of which the proof can be found in derivation S1. We formally extend BPTT to continuous time for a number of types of dynamical systems. Continuous time variants of BPTT have been considered before (e.g., [18]), but these derivations focus only on neural network-like systems and start from an Euler approximation of differential equations. We derive continuous time BPTT directly, without the need for approximations, and in a generic form which is applicable to a much wider variety of dynamical systems.

#### Instantaneous gradient

We consider continuous-time dynamical systems characterized by a state 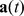 and a set of parameters 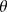, that is excited by an external input signal 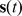. For brevity, the following summary focuses on ordinary differential equations (ODEs), i.e.:
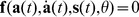
(1)


The derivations in the supplementary material are also given for the cases of delayed differential equations and for (delay) differential algebraic equations (one of our example systems, the photonic network, is described by a delayed differential algebraic equation).

Suppose that, at time 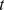, we wish to minimize the cost function 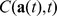. The gradient of this cost function w.r.t. the parameters, 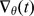, provides the direction in which the system parameters need to be changed to decrease the cost function at each point in time. It is given by
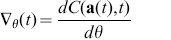


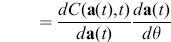


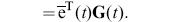



Here, 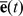 is the output error, i.e. the gradient of the cost function w.r.t. the system state 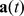), and 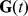 is the total derivative of the state 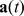 w.r.t. the parameters. For notational reasons we also introduce 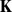, 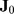, and 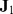, the partial derivatives of 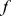 w.r.t. 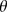, 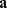, and 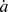, respectively. If we take the derivative of equation 1 w.r.t. the parameters, we can write:
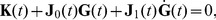
(2)Which is a differential equation that defines the evolution of 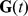, provided that 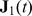 is an invertible matrix. This is for instance the case for explicit ODEs, where 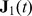 is the identity matrix. As 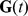 evolves according to an ODE, it can be computed in parallel with the system itself, and 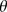 can also be updated continuously. This is known as *online learning*, as the system optimization happens while the simulation runs.

One downside of the above approach is that the matrix 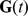 can be very large, as its number of elements equals the product of the number of state variables and the number of parameters in the system. Even for modestly sized dynamical systems, this number can grow into the several thousands, leading to a high computational cost.

#### Gradient time integral

One approach to drastically reducing the computational cost is called batch learning. Instead of continuously updating the parameters, one now considers a finite time interval of duration 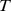 and defines the total cost as the time integral of 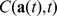 over this interval. We obtain the following expression for the gradient w.r.t. the parameters.
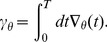
(3)


In this case, it is possible to avoid explicitly computing 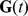 (see derivation S1). Instead, we need to solve a second system of differential equations that expresses the evolution of the error *backwards in time*:
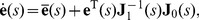
(4)in which 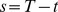. The gradient can then be replaced by



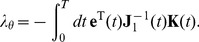
(5)This expression has two important advantages over the previous one. First of all, 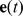 has the same dimensionality as 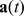, and hence the cost of computing 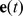 is roughly the same as that of solving the system itself. The second advantage is that for many systems, the evolution of individual state variables only depends on a small fraction of the parameters 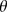. This is particularly the case in systems associated with sparse networks of interacting parts. As a result, 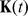 is often a very sparse matrix, and the multiplication in the integral can be solved efficiently. One important downside of this approach is the fact that it requires storing the time traces of the system state over the full interval 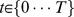. In general, it is advisable to keep the batches short. However, in some cases long batches are required, which may cause memory problems.

The above equations for computing 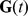 and 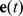 are the continuous-time equivalents of the machine learning techniques known as *Real-Time Recurrent Learning* (RTRL), and *Backpropagation through time* (BPTT), respectively. As stated in the introduction, equations ?? and 5 and the associated differential equations for 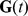 and 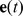 also appear in the theory of optimal control (for a comprehensive overview, see, e.g., [19]), in particular in modern, large scale applications which can only be solved numerically (e.g., as presented on the second SADCO industrial workshop 2012). In order to obtain good control solutions, a common approach is to write the input signal 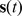 as a function of a finite set of parameters (often by means of interpolation or splines), and to optimize this discrete set of parameters in a way that is largely equivalent to the previously presented method to compute the gradient w.r.t. the system parameters. Here too, in order to find this gradient, additional differential equations need to be solved (known as costates), and they are essentially identical to those for 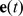 and 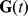 defined above. This intimate connection between backpropagation through time and optimal control has been described before [20], but has received little attention in further research.

### Numerical Simulations

We demonstrate our approach by embedding certain desired behaviors into simplified models of three different physical dynamical systems. Ordered according to increasing system complexity, they are: two-dimensional mass-spring-damper (MSD) networks, a beam of charged particles influenced by magnetic fields, and a photonic network of optical amplifiers. All details concerning the physical models and optimization methods can be found in the materials and methods. The presented optimization strategy can readily be used on more complicated and realistic simulation models, such as those used in the emerging field of simulation-based engineering [21].

#### Mass-spring-damper systems

We optimize MSD networks consisting of point masses connected with massless linear springs and dampers. The state of this system consist of the positions and velocities of the point masses. Possible optimisation parameters in this system are the rest lengths of the springs, the spring constants and the spring damping constants, as well as any parameters of possible control signals driving the springs.

**Figure 1:**
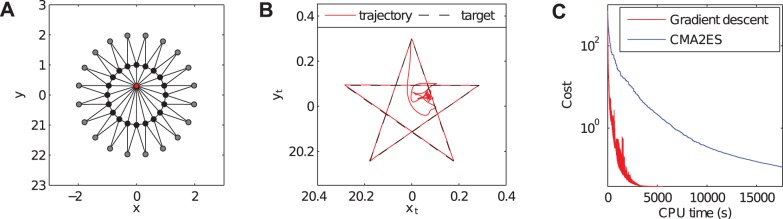
Embedding a trajectory in an MSD-network. **A:** Depiction of the initial condition of the MSD-system. The grey circles have fixed positions, the black circles are point masses that are non-fixed, and the red circle is the point mass of which we wish to control the trajectory. The connecting lines represent massless linear spring-dampers. **B:** Illustration of the trajectory of the selected node after optimisation. The full blue line is the actual trajectory, (including the trajectory after completing the pentagram) and the dashed line is the target. **C:** Comparison of the convergence speed of gradient descent and CMA-ES for the same initial parameter set.

First, we embed a specific trajectory: when the network evolves dynamically from a predefined initial condition, one of the nodes has to trace a pentagram shape. In order to achieve this, no external control signal is applied and we only optimize the rest lengths and spring constants of the springs. The optimized MSD network tracks the pentagram with nearly perfect accuracy, demonstrating BPTT's ability to find a solution for such tasks. The simplicity of this task (no noise; a single, well defined objective function) allows for a direct comparison with the so-called *Covariance Matrix Adaptation Evolution Strategy* (CMA-ES) [22], one of the most widely used evolutionary algorithms. The shape of the setup and the resulting trajectory are shown in Fig. 1a and 1b, respectively. Fig. 1c compares the convergence speeds of CMA-ES and gradient descent for this task, and shows that gradient descent converges substantially faster. This is to be expected, as CMA-ES makes an estimate of the gradient from multiple samples, whereas we compute it directly. This particular problem only has 160 parameters, which is still feasible for CMA-ES. As dimensionality increases, evolutionary algorithms will face increasing difficulty, as the cost of sampling the search space increases exponentially.

**Figure 2:**
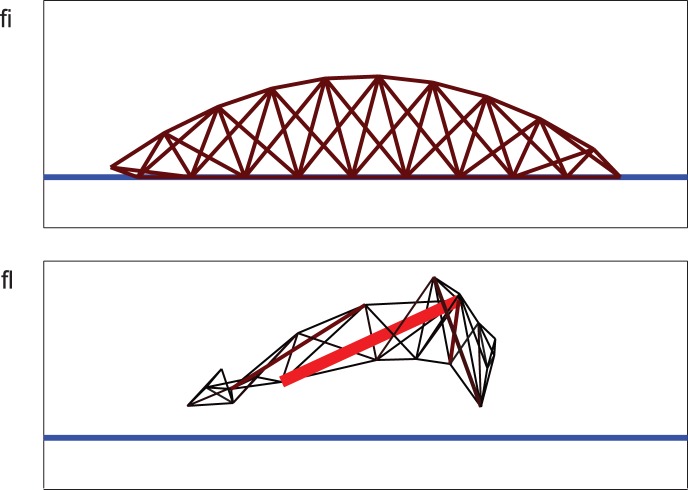
MSD robots. We have represented the springs that constitute the MSD robots with lines, where we visualized their modulation amplitude by making springs with large modulation amplitudes thicker and redder. The blue line represents the ground. **A:** Shape of the robot at the initialization of training. All springs have an equally large modulation amplitude. **B:** Snapshot of an example robot after finishing training. Only 4 of its springs still have a non-negligible modulation amplitude, and they provide virtually all locomotive power.

Second, we optimize an MSD model for locomotion, a problem which has often been studied in the context of evolutionary algorithms [6], [23] and matches well with the concept of morphological computation [6]–[8]. Our ‘robots’ are MSD-networks of which the spring resting lengths are modulated periodically. They exist in a 2D environment with gravity and a ground contact model. Initially, the robot is a worm-shaped set of springs of which each spring's rest length is periodically modulated with a random phase and amplitude. In this application, we optimize both the robot's shape (the spring rest lengths) and its control (the phases and amplitudes of the modulation). As the number of parameters for this problem is still manageable (162 in total), we use the online learning approach, which allows us to gradually optimize the robot while the simulation runs. The cost function consists of two contributions. The first is simply the squared difference between the average horizontal speed and a target value. The second is the sum of squares of the rest lengths and amplitudes in the cost function. This is added to avoid the trivial solution of ever increasing the modulation amplitudes and the rest lengths of the springs (which leads to larger contractions and a greater speed). Note that this second cost term is equivalent to the ubiquitous L2 regularization strategy in machine learning, where it is used to avoid extreme parameter sensitivity, and hence overfitting. We show a schematic depiction of the initial robot and an example of a trained robot in Fig. 2.

Videos of an initial (unoptimized) MSD robot (movie S1) and of some resulting optimized locomotions (movies S2–S4) are available in the supplementary material. The robots develop highly dynamic gaits in which usually only the front and rear extremities touch the ground. Even though the initial shape of the robot is identical for all experiments, the initial parameters that determine the control (in particular the phase of the periodic modulation), are chosen randomly at the beginning of each simulation. These small differences do lead to strongly differing final robot morphologies and gates, indicating that this problem has a high number of local optima.

Interestingly, when examining the parameter values for the modulation amplitudes, it appears that, due to the imposed restrictions on size and strength, the robots tend to end up with only a few springs with a large modulation amplitude, providing the bulk of the locomotive power, whereas the other springs exhibit small to virtually zero amplitudes. This poses an interesting possibility: when one would actually design and build a physical robot, it would be desirable to have as little actuated parts as possible. One could then use L1 regularization [24], which leads to a sparse solution in which a large part of the amplitudes are zero (which would greatly simplify the eventual robot construction).

#### Magnetic focusing

**Figure 3:**
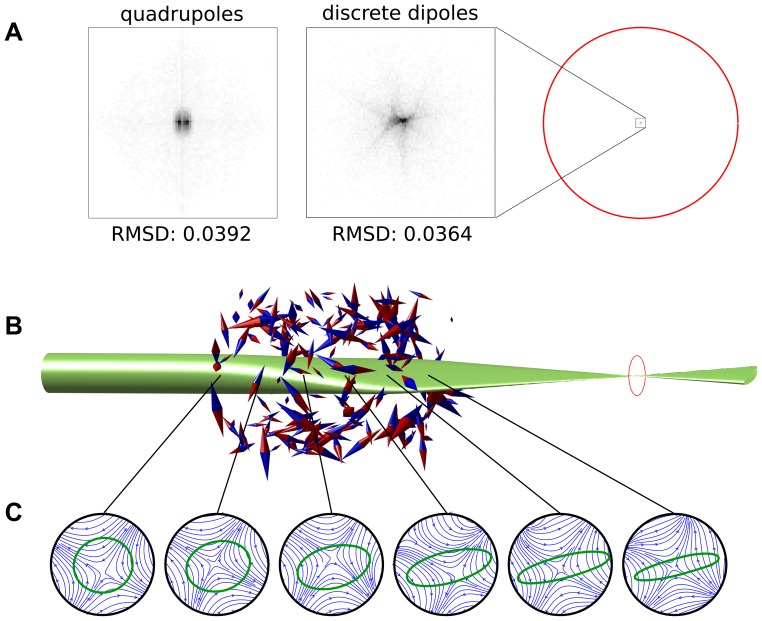
Illustration of the magnetic beam focuser. **A:** Particle distribution in a cross-section of the focal plane, shown for 50,000 particles, both for a set of two ideal quadrupoles and our configuration (CT-RTRL results). The cost function, the root mean squared distance (RMSD) of the particles w.r.t. the focal point as they pass through the focal plane, is shown underneath the panels. On the right we show the scale compared to the original beam width (red circle). Note that the spread of the particles for the quadrupoles is largely due to a relatively large spread in particle velocities. **B:** Illustration of the particle beam envelope (light green) and the spatial configuration of magnets (blue red cones), indicating position, direction and magnitude. **C:** Cut-through illustrations of the particle beam envelope and the lateral magnetic field at different positions throughout the beam focuser.

The MSD networks from the previous section are highly simplified, and cannot directly be constructed physically without taking into account a range of more realistic effects such as nonlinear springs, contacts and collisions. In the next example we have trained a spatial configuration of magnets to focus a beam of charged particles, which is a more practically applicable physical design problem. This problem has a well-known solution, consisting of two ideal quadrupole fields placed behind each other at a 90 angle [25]. Producing an ideal quadrupole field, however, requires a precisely manufactured geometry of magnetic cores. We use a discrete set of 200 point dipole magnets (leading to 1,200 trainable parameters: all magnet positions and orientations, having 3 coordinates each), which cannot produce such a field exactly. As such, gradient descent needs to find an approximate solution to the problem. In order to use the presented framework, we simulate an incoming ‘beam’ of particles (a discrete number of them), and use their positions and velocities as the state of the dynamic system. The resulting magnet configuration, beam, and distribution of particles crossing the focal plane are shown in Fig. 3, as well as a set of cross-sections of the beam within the lens, showing the lateral magnetic field lines within. The configuration manages to focus the beam with slightly better focus than the quadrupole set we compare against. Interestingly, the shape of the beam and the cross-sections show that the magnet configuration has found a solution that is qualitatively similar to that of an ideal quadrupole lens, in which the beam is first focused in one direction and then in the other.

#### Photonic networks

The third dynamical system we consider is an integrated network of semiconductor optical amplifiers (SOAs). Nonlinear photonic networks have been considered as promising candidates for information processing [26]–[29]. Essentially, the dynamics of networks of photonic components can show parallels with those of recurrent neural networks, making them an interesting platform for integrating high-bandwidth neural-network-like systems in physical hardware. SOA networks like the ones in [27], [30] are an interesting example of our technique because there exist non-negligible interconnection delays between the different amplifiers. Due to the finite speed of light and the fact that the internal dynamics of the SOAs are extremely fast, these need to be taken into account explicitly, and influence the system dynamics in a meaningful fashion. The model that describes this SOA network is a delayed differential algebraic equation.

In this example, we show that our approach can handle uncertainty due to manufacturing variations and noise, yielding robust and manufacturable designs. We use gradient descent to optimize the parameters of a 4
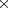
4 network of SOAs, interconnected with optical waveguides, inspired by earlier work [30]. The dynamical model we use for the individual SOAs has been shown to be an excellent approximation of reality [31]. The optimisation parameters for this system are the bias currents of the SOAs the losses and phase changes of all input and inter-SOA connections, as well as the delays of the inter-SOA connections. The desired output is realised by linearly combining a fraction of the light coming out of the SOAs and converting this to the electrical domain using a photodetector. Hence, the losses and phase changes in the readout connections are also optimised. On-chip photonic interconnections are etched from a silicon substrate with a finite resolution. This causes small variations on the exact length of the connections, and hence the phase of the light arriving at each SOA. In addition, each SOA produces a certain amount of noise in the form of amplified stimulated emission. Again, we need to include this noise in the optimization algorithm in order to obtain a robust solution. Both phase variability and noise were included in our optimizations.

**Figure 4:**
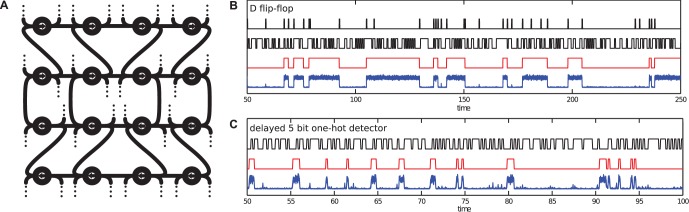
Results for optimised SOA networks. **A:** Schematic representation of the SOA-network. The black lines are photonic waveguides. The circles are the SOAs, with the arrows indicating in which direction the light passes through. Each SOA receives an external input signal, and at its output, a channel goes to the output (represented by the dotted lines). These channels are optimally combined optically and the output signal is the optical power of this combination. **B:** Illustration of the photonic D flip-flop. The top two time traces show the two input channels, the clock (set) signal and the data signal. The red time trace is the desired output power of the SOA network, and the blue one is a superposition of the measured output power for 10 different instances, each with different noise and internal phase variations. **C:** Illustration of the photonic 5-bit one-hot detector. The black time trace is the input signal, a random bit stream, and the red time trace is the desired output power of the SOA network. The blue one is a superposition of the measured output power for 10 different instances, each with different noise and internal phase variations.

We have optimized the network twice, once to behave like a photonic D flip-flop and once to realise a 5-bit delayed one-hot detector (a device which should produce an output spike when exactly one of the past five bits of the input stream was equal to one). In order to train the networks we use input/output example time traces, and an associated cost. In order to obtain robust designs, we took care to provide enough and sufficiently diverse training examples. In particular, the D flip-flop was not trained with a periodic clock to avoid solutions that internalize the clock period in the internal delays and do not work properly for other clock periods. The one-hot detector was trained to operate at a single clock frequency, but it did not receive a clock input, which means that it had to extract the clock phase itself. Due to the fact that we optimize this system by randomly sampling input/output examples, we essentially train this system using *stochastic gradient descent*, which is currently one of the most popular training methods in machine learning problems that involve large amounts of data [32]. A schematic depiction of the SOA network and example time traces for the trained networks are shown in Fig. 4, showing that despite substantial levels of noise and the included manufacturing variations, gradient descent is capable of training the networks to nearly perfect accuracy for both tasks (see supplementary methods for more detailed performance measures). Note that a photonic flip-flop can in fact be constructed with significantly fewer components [33], [34]. We use the example here to show that the presented method is capable to create a working solution automatically from nothing more than input/output examples and an associated cost function, and indeed that the SOA network is generic enough to embed several different behaviors within its parameters.

## Discussion

In this work, we have shown that it is possible to optimize surprisingly complex dynamics within a range of physical systems, by extending backpropagation through time to continuous time. Our examples illustrate that energy-efficient, robust and manufacturable solutions can be achieved by applying some of the knowledge that has been built up in the machine learning community.

First we have considered mechanical MSD systems. Using an online variant of the BPTT algorithm, we were able to automatically find energy efficient solutions for locomotion in simulated MSD robots. Despite the fact that the obtained gaits are fast, natural and efficient, the control of the optimized robots is extremely simple (periodically modulated rest lengths of the involved springs), and the full motion emerges synergistically between the control parameters and the robot's shape. This shows that our extension of BPTT can be very useful in the field of morphological computation and embodiment [7].

Second, we have configured a set of dipole magnets in order to focus a beam of charged particles, obtaining a solution that is in many ways equivalent to a known solution to this problem: a double quadrupole field. This example shows that BPTT can be useful in finding non-trivial solutions for problems that occur in designing electromagnetic devices, and perhaps even plasma physics.

Finally we have applied BPTT to a realistic example from the domain of photonics. We have optimized the internal parameters of a network of integrated SOAs and waveguides in order to make it perform two digital operations on input streams. In this case, the model for the system dynamics was more complex than in the previous systems, as it required delayed differential algebraic equations. In addition, to obtain robust and manufacturable solutions, we included realistic levels of parametric variations and system noise directly into the training process. Yet, despite the increased complexity of these examples, our approach has succeeded in automatically finding highly performant and robust solutions for both tasks.

In all these instances it is clear that BPTT is able to truly exploit the dynamic part of the system, and automatically link events that are separated in time. For instance in the case of the magnetic beam focuser: information of the objective function is only available when particles reach the focal plain, yet at that point in time, their interaction with the magnets has already happened. Due to the way BPTT takes into account the state history, it still provides a solution to the given problem. Similar in the case of the photonic flip flop: the on/off state is remembered indefinitely long, and that means that internally, the network has produced two stable point attractors between which it can toggle only when a clock pulse arrives. This indicates that BPTT can manipulate the dynamics of the system in a profound way.

The applicability of gradient descent to real-world dynamical systems greatly depends on the accuracy of existing models and on the understanding of associated stochastic phenomena such as noise and process variation. In the fields of electronics and photonics, highly accurate models exist, but variability can be considerable. Other domains, such as, e.g., robotics, are known to suffer from a lack of accurate models. Applying BPTT for such problems poses a challenge. Conceivably, in some cases even an approximate model could provide a useful gradient and help to identify approximate parameter values which can subsequently be fine-tuned using more accurate simulations. When an analytical model is not available, an approximate model can be built from measurements or simulations, e.g. using self-modeling approaches [35]. Further research will need to be conducted to find if such a strategy is feasible or not.

The ability to embed specified dynamic behaviors into a generic physical platform opens a broad range of possible applications. For example, it may provide a large jump in research concerned with bringing computation outside the silicon domain. Designing physical devices that robustly and efficiently perform non-digital computations now become feasible. The proposed methodology can also be used by engineers to design machines and robots that exploit their inherent non-linear behavior in ways that were previously too difficult to explore. It could even be extended to embed non-trivial dynamical behaviors into passive or active continuous media which are characterized by partial differential equations, since the mathematics for controlling such systems already exists [36]. This could lead to e.g., systems that facilitate the task of sensors, chemical controllers based on reaction-diffusion systems [37]. Our technique could also help to gain more insight into the cost functions that were optimized by nature in complex biological systems by emulating them.

One important contribution of this work is that it fades the boundaries between several disciplines and provides part of a roadmap towards integrating optimal design, machine learning, and optimal control into one discipline. Researchers working on designing complex physical systems would not easily consider machine learning as a potential optimization strategy. On the other hand, the machine learning community generally considers the computer the only platform on which to implement their models. Paul Werbos, who is often considered as the originator of the backpropagation algorithm [12], [38] through his framework of *ordered derivatives*, has already pleaded for a better cooperation between scientists and engineers in these fields and others (most noticeably *automated differentiation*) [39]. The main difficulty in the application of our technique lies in the derivation of the matrices 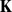, 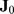, and 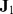, which can be tedious and needs to be done for each new system. The potential of such an interdisciplinary collaboration lies in the creation of machine-learning based automated design tools for generic dynamical systems, in which only the system equations and the examples need to be provided by the user. We hope that this paper can pave the way for the realization of this vision by combining the necessary mathematical and machine learning background and by providing convincing design examples.

## Materials and Methods

### Notes on Gradient Descent

Dynamic systems can exhibit bifurcations, which are associated with very rapid changes in dynamics as a function of the parameters [40]. This translates to extremely steep parts in the cost function and as a result, very large gradients. Simply updating the system using this gradient will lead to a very large and unpredictable change of the parameters, and may in fact break down the training process altogether. In order to deal with this, we normalize the gradient before using it for parameter updates, essentially only the direction of the gradient and not its magnitude.

We use two strategies: online (CT-RTRL) and batched training (CT-BPTT). In the online case, parameter updates happen continuously. In other words, 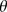 will depend on time, and evolves according to:
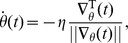
Here we need to make sure that the time scale at which 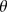 changes is much slower than the timescales of the DS, otherwise the training process will interfere with the actual dynamics of the system. In the batched training, parameters are updated offline in between discrete simulation instances of a fixed length. The update equation can be written as:
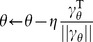



In all our experiments we use dimensionless units for simplicity. In the final example of photonic networks, however, we will use values that reflect realistic physical values of SOA parameters. Often, the numerical scaling of different kinds of parameters (e.g., spring constants vs. their rest lengths) may differ orders of magnitude. If this is the case, we will normalise their respective gradients separately and set separate learning rates.

In order to ensure convergence we let the learning rate decay over the course of the experiments (in either a linear or an exponential fashion).

### Notes on Implementation

All experiments shown in this paper were performed on a single laptop computer with 8 GB RAM and a 2.3 GHz Intel Core i7 processor. We used Matlab for our experiments and made the code for generating the results available on http://users.elis.ugent.be/~mhermans/code.zip.

### Experimental Details

#### Embedding a trajectory in an MSD-system

The state of the system is made up of the mobile node positions and velocities. The force exerted on the 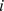 -th node, exhibited by a spring connecting the 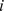-th and 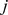-th node is equal to
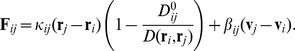



Here, 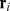 and 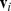 represent the position and velocity of the 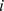-th node respectively. The parameter 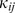 is the spring constant for this particular spring, 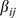 is its damping constant, and 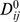 its rest length. 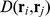 is the euclidean distance between the 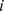-th and 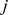-th node.

At 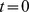, the springs are allowed to relax, and the centre mass will follow a certain trajectory that is determined by the system parameters. We have optimized the rest lengths and spring constants (where we made sure these could not grow smaller than zero by truncating their values).

The target trajectory has the shape of a pentagram. To avoid instantaneous changes in velocity on the corners of the star we made sure that the velocity of the desired trajectory goes to zero at the turning points. In practice: if one straight segment of the pentagram is traced over a time interval 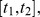 then its speed evolves as 
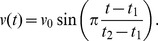
 Once we have constructed the target position as a function of time, we derive the accompanying velocity by taking its derivative, and use this velocity as an additional target for the central node state.

- **Cost function** The cost function is the sum of the mean square errors of the position and velocity of the target node.
- **CMA-ES details** The comparison with CMA-ES was made using a standardized implementation, available at https://www.lri.fr/~hansen/cmaes_inmatlab.html. The only parameters which need to be set by the user are the population size and the initial standard deviations of the parameters. Since we know that the relative scaling of spring constants vs. rest lengths is about a factor 25, we also set the initial values for parameter standard deviations accordingly (25 times greater for the spring constants than the rest lengths). We found that a tradeoff between good performance and speed of convergence was found with a quite small populations (20 individuals, small compared to the dimensionality of the problem), and small initial standard deviations (0.04 for the rest lengths and 1 for the spring constants). Using these parameters, we ran 5 experiments and chose the best end result to compare with gradient descent. Note that gradient descent has no stochastic element in this case, such that we only needed to run one experiment to obtain the result.
- **Gradient descent details** We used CT-BPTT with batches of length 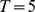, the time needed to complete the target trajectory. We optimized the learning meta-parameters; the initial learning rate and the rate at which it decreases after each training iteration. We normalized the full parameter gradient (not separately for the two parameter sets), and chose an initial learning rate of 5 for the spring constants and 0.05 for the rest lengths. Each training iteration both learning speeds were multiplied with a factor 0.999.
- **Implementation details** We used leapfrog integration for the forward simulation and Euler integration for the error backpropagation. The step size was chosen at 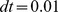.
- **Initial parameters** Initial spring constants were set to 25. Damping constants were all equal to 0.1, and the rest lengths were chosen as the distances between the nodes when the system is in its initial condition, but with the target mass in the centre.

#### MSD robots

For the locomotion experiment, each spring's instantaneous rest length is given by
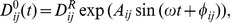
in which 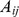 and 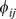 are amplitude and phase respectively, and 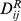 is the rest length without modulation. The exponential function assures that the modulation signal cannot become negative, yet reach high peak values if desired. We use a highly simplified model for the ground, with an upward force 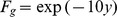, such that it is nearly zero above ground (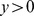), but increases very rapidly below ground. Ground friction only acts in the 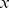-direction, and is modeled as 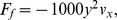 if 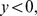 and 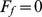 if 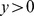 As such, the harder a node is pressed down, the stronger lateral friction will be.

- **Cost function** The cost function is the mean square error between the target velocity and the mean robot velocity in the 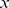-direction. Additionally, we add the sum of squares of the amplitudes and the rest lengths, scaled with 0.2 and 0.001, respectively. The target speed increases slowly over time as 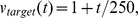 such that the robot slowly speeds up during training.
- **Training method** We used online training, where the initial learning rates for amplitudes, phases, and rest lengths are 0.05, 0.2, and 0.2, respectively, and the corresponding gradients are normalized separately. Due to the constantly changing target velocity, learning rates are kept constant over time.
- **Implementation details** We used leapfrog integration for the forward simulation and Euler integration for updating 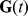. The step size was chosen at 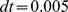.
- **Initial parameters** Spring constants were all equal to 100. Damping constants were all equal to 1, and the rest lengths were chosen as the distances between the nodes when the system is in its initial shape. Amplitudes were initialised at 0.2, and all phases were picked randomly between 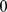 and 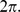

#### Magnetic lens

In order to simulate the beam, we used a set of 200 discrete, non-interacting particles passing through the lens. All particles are initialized on a uniformly sampled position in a circle with radius one, (the source), and their initial velocity is always aligned with the beam, and has mean value 4 and standard deviation 0.1. If a particle crosses the focal plane, or its distance to the beam axis is greater than 2, it is reinitialized at the beam source (and its corresponding 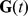 is reset to zero). This way, the beam remains constantly present during the training phase.

The beam is lying along the 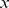-axis, the source is at 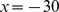, and the focal point at 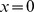. All magnets positions are bound such that their distance to the beam axis (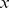-axis) is no smaller than 2, and their 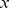-coordinates lie between 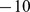 and 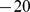. Their magnetic moment 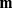 has a magnitude capped at 10, but in practice none even come close to this bound.

Each particle has unit charge and mass, and feels the magnetic Lorentz force: 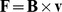. The magnetic field at each location is the sum of the fields of each magnetic dipole. A single magnetic dipole with magnetic moment 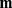, located at the origin, has a magnetic field given by
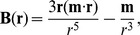
where we omitted the scaling factor 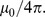


We used 200 dipole magnets, leading to a total of 1200 parameters (3 coordinates for magnet moment and 3 for position).

- **Cost function** The cost function is equal to the mean square error between the particles velocity and that pointed towards the focal point. The magnitude of the target velocity is that equal to the initial particle's velocity (static magnetic fields cannot change the velocity of a charged particle, only its direction.) The resulting output error 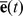 is next scaled for each particle with a factor 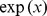, such that only particles close to the focal point actively contribute to the overall cost.
- **Quadrupole lens** In order to make the comparison we implemented a simulation in which two ideal quadrupole fields were placed at 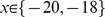 and at 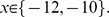 In these regions the magnetic field components are given by 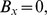 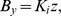 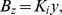 in which 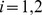 for the two fields. Everywhere else the magnetic field was equal to zero. Parameters 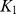 and 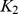 were optimised using the mean square distance to the focal point when the particles cross the focal plane. We used a brute force search for optimisation.
- **Training method** Training is performed online. We used a learning rate that linearly decays over the course of the experiment, which runs for a time 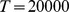, with an initial value 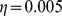.
- **Implementation details** We used leapfrog integration for the forward simulation and Euler integration for updating 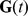. The step size was chosen at 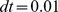.
- **Initial parameters** All initial dipole 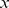-coordinates were uniformly sampled between the bounds described above. Their distances to the beam axis were chosen between 2 and 4, and their angle w.r.t. the 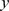-axis randomly sampled between 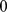 and 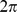. Magnetic moment coordinates were chosen from 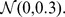

#### Photonic SOA networks

Each SOA has an internal state 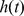 which describes the SOA gain. It evolves according to
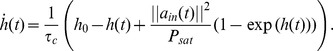



Here, 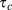 is the free carrier lifetime, 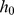 is the rest value for 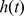, which is determined by an external bias current, 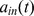 is the complex field at the SOA input side and 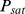 is the saturation power of the SOA. We chose 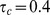, 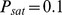. The field that exits the SOA is described by
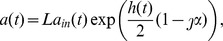
where 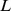 is a factor corresponding to internal losses (which we chose fixed at 0.5), 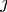 is the imaginary unit and 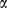 a constant depending on the SOA which we chose at 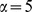. For further details and the derivation of these formulas we refer to [41].

The incoming field for a given SOA within the network is given by
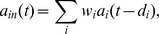
where 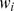 is the complex weight associated with a single connection (described in more detail further in this section), 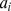 are the complex fields of all other SOAs and input channels that connect to this SOA, and 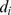 are the associated connection delays. We optimised the following parameters: the complex weights 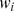 of each SOA, the delays 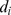, and the bias current 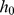 for each SOA, leading to a total of 152 parameters for the one-hot detector, and 184 for the flip-flop (counting the real and imaginary parts of the complex weights as separate parameters).

The delayed 5-bit one-hot task input consisted of a random bitstream with period 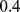. The flip-flop input existed of one random bitstream (the data), with period 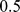, and a clock input, where at each time a new data bit enters the network, there's a one in ten chance of a clock pulse. The clock pulse has length 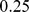.

Each interconnection will introduce a fixed phase shift and a decrease in amplitude, which can be combined to a single complex weight with which the complex amplitude is multiplied. Additionally, each interconnection will have a certain delay value. If we assume that at the output side of each SOA half the power goes to the output connection, and the remaining fraction is split in two as it connects to at most two other SOAs in our particular network architecture, we can state that only at most a quarter of the output power from one SOA reaches another SOA, which means that the moduli of the complex weights are truncated at 0.5. The values of the delays were bounded between 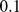 and 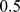 for the one-hot task, and between 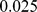 and 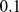 for the flip flop task. Note that in principle the delay and the phase shift of a connection are codetermined by the physical length of the connection. In practice, however, the wavelength is much shorter than an interconnection, such that even a tiny shift in length will cause a very large phase shift. Therefore we consider these two parameters as independent.

The precision at which the phase shift of an interconnection is manufactured is determined by the precision at which connection lengths can be made, and hence also dependent on the wavelength of the light. In order to model these variations, each training iteration we perturb the phases of all interconnections by adding them with a random phase, sampled from 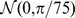 (based on manufacturing precision with standard deviation of 10 nm and an optical wavelength of 1550 nm), and use the according weights to compute the gradients on. This assures that the found solution is not extremely sensitive to small variations in phase.

Finally, we superpose noise on each SOA's output field, modeled as a mixture of frequencies near that of the signal frequency based on [42]. The noise has the following time dependence:
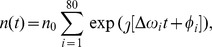
where 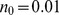, and 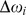, and 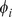 are values which are sampled randomly at each training iteration between from 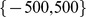 and 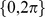, respectively. This noise takes on the form of a quickly fluctuating signal with a low amplitude (its power being roughly 50–100 times lower than the average output power of the SOAs after training). Each SOA node receives both network feedback and an external input signal, which consists of a weighted sum of input data channels and a constant bias signal. The corresponding connections we have modeled to have zero delay. The output light signal is constructed by combining the output light of each SOA. Here again, each of these output connections has an associated complex weight, and we again assumed they have no delay.

- **Cost function** In the considered tasks we are only concerned with output power, not phase. Therefore the cost function was the mean square error between the desired and actual output power (which for both tasks vary between zero and one).
- **Training method** Training is performed in batches of 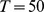 and 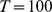 for the one-hot and flip-flop task, respectively. Each training example was made from a randomly sampled bitstream, such that optimization of the network is performed with stochastic gradient descent. The learning rate was started at 0.1 and reduced with a factor 0.9999 per training iteration. As soon as performance became adequate (by visually inspecting the results), we set this factor to 0.999 for quicker convergence. The trained parameters include all weights, the rest gain 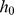 of all SOAs, and all delays. The weights and rest carrier densities gradients were normalized separately.
  Training delays posed a practical difficulty, as in our simulation delays are still represented by a discrete number of time steps. Therefore, we only considered the sign of the respective gradients and either increased or decreased them with one time step each update. We are not certain whether training the delays poses a significant help in this case, especially since the delay gradient depends on the time derivatives of the SOA outputs, which have been polluted with amplified spontaneous emission noise.

- **Implementation details** We used Euler integration with time step 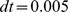. Delays were implemented by picking corresponding SOA output values from recorded time traces at a discrete number of time steps in the past.
- **Initial parameters** All weights of all connections were initialized with phases sampled uniformly from 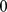 to 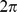. Initial moduli of internal connections were sampled uniformly between 0 and 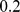, those of input data between 0 and 0.25 for the flip flop task and between 0 and 0.5 for the one-hot task. Input bias weights were initialized between 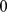 and 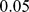, and initial output weights all had modulo zero. Delays were uniformly sampled between their minimal and maximal value. The rest gains were initially all chosen at 3.
- **Validation** To measure how well the trained networks performed, we measured ROC curves in two ways: by taking the average output power over the duration of an entire bit, or by sampling output power at the last time step of a bit. For each task we measured this for 10 sequences of 50000 time steps, each with different input sequences, phase variations and noise. We omitted the first 10 bits as the networks may still be in a transient state from initial conditions. The ROC curves themselves were nearly perfectly square, so we do not show them. Instead we measured the area-under-curve (AUC) (equivalent to bit error rate), which was exactly equal to one for the photonic flip flop, for both methods of measurement (perfect performance for the given number of test instances). The one-hot detector had an AUC equal to exactly one when taking the average output power over each bit, and 0.9999 when using the last sample of each bit.

## Supporting Information

Movie S1 **Animation of the MSD robot before optimisation commences.** The blue horizontal line represents the ground, black lines are massless springs and the coloured dots are point masses (coloured for distinction).
(MOV)

Movie S2 **Animation of an example outcome of an MSD robot after optimisation.** The blue horizontal line represents the ground, black lines are massless springs and the coloured dots are point masses (coloured for distinction).
(MOV)

Movie S3 **Animation of an example outcome of an MSD robot after optimisation.** The blue horizontal line represents the ground, black lines are massless springs and the coloured dots are point masses (coloured for distinction).
(MOV)

Movie S4 **Animation of an example outcome of an MSD robot after optimisation.** The blue horizontal line represents the ground, black lines are massless springs and the coloured dots are point masses (coloured for distinction).
(MOV)

Derivation S1 Mathematical derivation of the BPTT algorithm for continuous-time systems.
(PDF)
