# Supplementary material for: Automated Design of Complex Dynamic Systems
Source: PLoS One. 2014 Jan 31;9(1):e86696. doi: 10.1371/journal.pone.0086696 (PMC3908928; doi:10.1371/journal.pone.0086696)
Supplement: Derivation S1 — Mathematical derivation of the BPTT algorithm for continuous-time systems. (PDF) [file pone.0086696.s005.pdf]

Automated Design of Complex Dynamical Systems: Supporting Derivations

Michiel Hermans, Joni Dambre, Benjamin Schrauwen, Peter Bienstman

January 8, 2014

1 Structure

This document contains the mathematical derivations for the paper: Automated Design of Complex Dynamical Systems. It is structured as follows. Section 2 is devoted to explaining the BPTT algorithm as it is commonly used for training recurrent neural networks in machine learning. It explains the discrete-time version of BPTT and the necessary concepts. Is section 3 we introduce the online version of continuous time backpropagation through time for ordinary differential equations. In section 4 we show how it is possible to transform this into a computationally less demanding algorithm. Sections 5 to 7 provide similar derivations for important other kinds of differential equations, such that the primary theoretical contribution of this paper is contained within sections 3 to 7. Throughout this document, we will often use the word 'training' in the same context as 'optimisation'.

2 Discrete time backpropagation through time

Before we start, it is useful to consider notations. Throughout the following derivations (sections 3 to 7), we will use bold uncapitalised letters for column vectors, except for the gradients $\nabla_{\boldsymbol{\theta}}$ and $\boldsymbol{\gamma}_{\boldsymbol{\theta}}$ which are row vectors by definition, and bold capital letters for matrices. When we define a derivative of the form $d\mathbf{x}/d\mathbf{y}$, this is a matrix with vertical size the dimension of $\mathbf{x}$ and horizontally that of $\mathbf{y}$.
In this section we will give a brief overview of backpropagation through time (BPTT) as it is commonly applied in recurrent neural networks (RNNs). Suppose we have a state $\mathbf{a}_t$, which is governed by the following update equation:

$$\mathbf{a}_{t+1} = \mathbf{f}(\mathbf{a}_t, \mathbf{s}_t, \boldsymbol{\theta}),$$

in which $\mathbf{s}_t$ is a discrete time external input signal, and $\boldsymbol{\theta}$ is the set of parameters that need to be updated. In typical recurrent neural networks (RNN), these parameters are the elements that make up a square recurrent weight matrix $\mathbf{W}$ and input matrix $\mathbf{V}$, and the nonlinear function is commonly a sigmoid function, e.g., the hyperbolic tangent. The equation takes the form

$$\mathbf{a}_{t+1} = \tanh\left(\mathbf{W}\mathbf{a}_t + \mathbf{V}\mathbf{s}_t\right),$$

but here we will keep the derivation fully general.
The goal is to minimise a certain cost function $C(\mathbf{a}_t, t)$ at a certain moment in time. The

gradient of this cost function w.r.t the parameters is given by

$$\begin{aligned}\nabla^t_{\boldsymbol{\theta}} &= \frac{dC(\mathbf{a}_t,t)}{d\boldsymbol{\theta}} \\ &= \frac{dC(\mathbf{a}_t,t)}{d\mathbf{a}_t}\frac{d\mathbf{a}_t}{d\boldsymbol{\theta}} \\ &= \bar{\mathbf{e}}_t^{\mathsf{T}}\mathbf{G}_t. \end{aligned} \tag{1}$$

Here, $\bar{\mathbf{e}}_t$ is the output error (the transpose of the gradient of the cost function w.r.t, $\mathbf{a}_t$), and

$$\mathbf{G}_t = \frac{d\mathbf{a}_t}{d\boldsymbol{\theta}}, \tag{2}$$

is the total state derivative w.r.t. $\boldsymbol{\theta}$. Due to the recursion relation, $\mathbf{a}_t$ will depend on $\mathbf{a}_{t-1}, \mathbf{a}_{t-2}, ..., \mathbf{a}_0$ (we assume that $\mathbf{a}_0$ is a fixed initial value). This means that we can use the chain rule to "unfold" the total derivative in $\mathbf{G}_t$ in time, until we end up with nothing but partial derivatives:

$$\mathbf{G}_t = \frac{\partial \mathbf{a}_t}{\partial \boldsymbol{\theta}} + \frac{\partial \mathbf{a}_t}{\partial \mathbf{a}_{t-1}}\frac{\partial \mathbf{a}_{t-1}}{\partial \boldsymbol{\theta}} + \cdots + \frac{\partial \mathbf{a}_t}{\partial \mathbf{a}_{t-1}}\frac{\partial \mathbf{a}_{t-1}}{\partial \mathbf{a}_{t-2}} \cdots \frac{\partial \mathbf{a}_2}{\partial \mathbf{a}_1}\frac{\partial \mathbf{a}_1}{\partial \boldsymbol{\theta}}. \tag{3}$$

If we define

$$\mathbf{K}_t = \frac{\partial \mathbf{a}_t}{\partial \boldsymbol{\theta}}, \tag{4}$$

and

$$\mathbf{J}_{t+1} = \frac{\partial \mathbf{f}(\mathbf{a}_t, \mathbf{s}_t, \boldsymbol{\theta})}{\partial \mathbf{a}_t} = \frac{\partial \mathbf{a}_{t+1}}{\partial \mathbf{a}_t}, \tag{5}$$

equation 3 can be rewritten as

$$\mathbf{G}_t = \sum_{i=1}^{t} \mathbf{Q}_{it}\mathbf{K}_i, \tag{6}$$

in which

$$\mathbf{Q}_{it} = \mathbf{1} \qquad \text{if } i = t,$$

and

$$\mathbf{Q}_{it} = \prod_{j=i}^{t-1} \mathbf{J}_{j+1} \qquad \text{if } i \neq t.$$

More efficiently, $\mathbf{G}_t$ can be computed with a recursion relation:

$$\mathbf{G}_t = \mathbf{K}_t + \mathbf{J}_t\mathbf{G}_{t-1}.$$

This forms the basis of real-time recurrent learning (RTRL), an online variant of the more common BPTT scheme. It provides a gradient for the associated cost at each time step, such that optimisation can happen during the system updates. The downside of this approach is that, while $\mathbf{K}_t$ is often very sparse, $\mathbf{G}_t$ generally is not, and computing it iteratively is slow and cumbersome compared to updating the recursion equation. It has dimensions equal to the number of states times the number of parameters, which can be a very large number.

Suppose that instead of computing the cost gradient at just one moment in time, we consider its sum over a whole sequence of length T:

$$\gamma_{\boldsymbol{\theta}} = \sum_{t=1}^{T} \nabla_{\boldsymbol{\theta}}^{t}.$$

Inserting equation 1 and replacing $\mathbf{G}_t$ with the expression given in equation 6 yields

$$\gamma_{\boldsymbol{\theta}} = \sum_{t=1}^{T} \bar{\mathbf{e}}_t^{\mathsf{T}} \sum_{i=1}^{t} \mathbf{Q}_{it}\mathbf{K}_i.$$

If we exchange the order of summation, we get

$$\gamma_{\boldsymbol{\theta}} = \sum_{i=1}^{T} \underbrace{\sum_{t=i}^{T} \bar{\mathbf{e}}_t^{\mathsf{T}} \mathbf{Q}_{it}}_{\mathbf{e}_i^{\mathsf{T}}} \mathbf{K}_i.$$

The variable $\mathbf{e}_i$ can be computed recursively, just like $\mathbf{G}_t$, but backwards in time (hence the term backpropagation through time):

$$\mathbf{e}_i^{\mathsf{T}} = \bar{\mathbf{e}}_i^{\mathsf{T}} + \mathbf{J}_i \mathbf{e}_{i+1}^{\mathsf{T}}.$$

This means that we can write the sum of the gradients over the whole sequence as

$$\gamma_{\boldsymbol{\theta}} = \sum_{i=1}^{T} \mathbf{e}_i^{\mathsf{T}} \mathbf{K}_i. \tag{7}$$

The advantage of this expression is twofold. First of all, computing the sequence $\mathbf{e}_i$ is usually comparable in computational cost to computing the sequence of states $\mathbf{a}_t$, as it has the same dimensionality. Furthermore, in most practical situations $\mathbf{K}_t$ is highly sparse. This is especially true for RNNs, but the argument remains valid for many continuous time systems as well (which is important in the following sections). The sum over matrix-vector multiplications in equation 7 can commonly be written as a single, highly efficient matrix matrix multiplication which only takes a small fraction of additional time to compute.
In what follows we will formally extend the presented algorithm to continuous time.

3 Cost gradient for ordinary differential equations

In this section we introduce the simplest method for computing a parameter gradient for dynamical systems (DS). Suppose we have a continuous-time dynamical system characterised by a state $\mathbf{a}(t)$ and a set of parameters $\boldsymbol{\theta}$. It is also excited by an external input signal $\mathbf{s}(t)$. The dynamics are governed by the following differential equation

$$\mathbf{f}(\mathbf{a}(t), \dot{\mathbf{a}}(t), \mathbf{s}(t), \boldsymbol{\theta}) = 0 \tag{8}$$

Suppose we have a cost function $C(\mathbf{a}(t), t)$ we wish to minimise. The gradient of this cost function w.r.t the parameters is given by

$$\begin{aligned} \nabla_{\boldsymbol{\theta}}(t) &= \frac{dC(\mathbf{a}(t), t)}{d\boldsymbol{\theta}} \\ &= \frac{dC(\mathbf{a}(t), t)}{d\mathbf{a}(t)} \frac{d\mathbf{a}(t)}{d\boldsymbol{\theta}} \\ &= \bar{\mathbf{e}}^{\mathsf{T}}(t)\mathbf{G}(t). \end{aligned}$$

Here, $\bar{\mathbf{e}}(t)$ is the output error (the gradient of the cost function w.r.t, $\mathbf{a}(t)$), and

$$\mathbf{G}(t) = \frac{d\mathbf{a}(t)}{d\boldsymbol{\theta}}, \tag{9}$$

is the total derivative of the state $\mathbf{a}(t)$ w.r.t. the parameters; a matrix with vertical dimension the number of elements in $\mathbf{a}(t)$, and horizontal dimension the number of elements in $\boldsymbol{\theta}$ For notational reasons we also introduce the partial derivative:

$$\mathbf{K}(t) = \frac{\partial \mathbf{f}(\mathbf{a}(t), \dot{\mathbf{a}}(t), \mathbf{s}(t), \boldsymbol{\theta})}{\partial \boldsymbol{\theta}}, \tag{10}$$

which has the same dimensions as $\mathbf{G}(t)$. Finally, we introduce the Jacobian of $\mathbf{f}$ w.r.t. $\mathbf{a}(t)$ as

$$\mathbf{J}_0(t) = \frac{\partial \mathbf{f}(\mathbf{a}(t), \dot{\mathbf{a}}(t), \mathbf{s}(t), \boldsymbol{\theta})}{\partial \mathbf{a}(t)}, \tag{11}$$

and w.r.t. $\dot{\mathbf{a}}(t)$

$$\mathbf{J}_1(t) = \frac{\partial \mathbf{f}(\mathbf{a}(t), \dot{\mathbf{a}}(t), \mathbf{s}(t), \boldsymbol{\theta})}{\partial \dot{\mathbf{a}}(t)}, \tag{12}$$

If we take the derivative of equation 8 w.r.t the parameters, we can write:

$$\begin{aligned} 0 &= \frac{\partial \mathbf{f}(\mathbf{a}(t), \dot{\mathbf{a}}(t), \mathbf{s}(t), \boldsymbol{\theta})}{\partial \boldsymbol{\theta}} + \frac{\partial \mathbf{f}(\mathbf{a}(t), \dot{\mathbf{a}}(t), \mathbf{s}(t), \boldsymbol{\theta})}{\partial \mathbf{a}(t)} \frac{d\mathbf{a}(t)}{d\boldsymbol{\theta}} + \frac{\partial \mathbf{f}(\mathbf{a}(t), \dot{\mathbf{a}}(t), \mathbf{s}(t), \boldsymbol{\theta})}{\partial \dot{\mathbf{a}}(t)} \frac{d\dot{\mathbf{a}(t)}}{d\boldsymbol{\theta}} \\ &= \mathbf{K}(t) + \mathbf{J}_0(t)\mathbf{G}(t) + \mathbf{J}_1(t)\dot{\mathbf{G}}(t) \end{aligned} \tag{13}$$

In other words, we have defined a differential equation for the evolution of $\mathbf{G}(t)$, provided that $\mathbf{J}_1(t)$ is an invertible matrix. This is for instance the case for explicit ODEs, where $\mathbf{J}_1(t)$ is the identity matrix. Computing $\mathbf{G}(t)$ allows to perform online learning. We can compute $\mathbf{G}(t)$ online, together with the actual simulation of the DS, and slowly adjust the parameters concurrently[1]. In reality, the dimensionality of $\mathbf{G}(t)$ may prove to be impractically large. Even though the size of the matrix is the same, the partial derivative $\mathbf{K}(t)$ is usually much sparser, as most variables only depend directly on a small subset of the parameters.

4 Offline backpropagation through time

Very much like in discrete-time RNNs, it is possible to avoid explicitly computing $\mathbf{G}(t)$. We can for continuous time let the error evolve backwards in time too, where it evolves according

[1] such that they change significantly slower than the relevant time scales within the DS

to a differential equation instead of an update equation. It is possible to get the same result by considering BPTT on an Euler integration of the differential equation, and taking the limit of $dt \to 0$. This step can be omitted, however, and we will stay in the continuous time domain, which proves that the validity of our approach does not depend on any specific method used to solve the differential equations. Let us start by considering the integral of the gradient for a given time span T. Indeed, if we consider limited time sequences in which we update the parameters slowly each moment in time using the previously defined gradient, the total change of the parameters would be the integral over time of the gradient (provided the change is so small that the dynamics of the DS are not changing during the considered time interval).

$$\begin{aligned} \boldsymbol{\gamma_\theta} &= \int_0^T dt \nabla_{\boldsymbol{\theta}}(t) \\ &= \int_0^T dt \bar{\mathbf{e}}^\mathsf{T}(t)\mathbf{G}(t), \end{aligned} \tag{14}$$

We will assume that the input signal, the internal state, the derivatives $\mathbf{G}(t)$ and the error signal $\bar{\mathbf{e}}(t)$ are identical to zero for $t < 0$ and $t > T$.
If we use $\boldsymbol{\gamma_\theta}$ to perform parameter updates, we can avoid computing $\mathbf{G}(t)$ explicitly. To achieve this, we introduce the variable $\mathbf{e}(t)$, which is governed by the following differential equation :

$$\frac{d\mathbf{e}(s)}{ds} = \bar{\mathbf{e}}(s) + \mathbf{x}(s), \tag{15}$$

where $s = T - t$, i.e., s is time running backwards, starting from T. This means that, in order to solve $\mathbf{e}(s)$ numerically, we need to integrate the equation backwards in time. The unknown variable $\mathbf{x}(s)$ is what we wish to find. Assuming that $\bar{\mathbf{e}}(t) = \mathbf{e}(t) = 0$ if $t < 0$ or $t > T$, we can implicitly solve equation 15 using an integral:

$$\mathbf{e}(s) = \int_0^s ds' \left[\bar{\mathbf{e}}(s') + \mathbf{x}(s')\right], \tag{16}$$

or in the normal time domain this becomes:

$$\mathbf{e}(t) = \int_t^T dt' \left[\bar{\mathbf{e}}(t') + \mathbf{x}(t')\right]. \tag{17}$$

As we will show, $\mathbf{e}(t)$ is the continuous time equivalent of the backpropagated error. We start by replacing $\mathbf{G}(t)$ in equation 41 by its implicit integral solution:

$$\boldsymbol{\gamma_\theta} = -\int_0^T dt\, \bar{\mathbf{e}}^\mathsf{T}(t) \int_0^t dt' \mathbf{J}_1^{-1}(t') \left[\mathbf{K}(t') + \mathbf{J}_0(t')\mathbf{G}(t')\right].$$

We can switch the order of integration over t and t', yielding

$$\boldsymbol{\gamma_\theta} = -\int_0^T dt \left(\int_t^T dt' \bar{\mathbf{e}}^\mathsf{T}(t')\right) \mathbf{J}_1^{-1}(t) \left[\mathbf{K}(t) + \mathbf{J}_0(t)\mathbf{G}(t)\right]. \tag{18}$$

If we reorder equation 17, we find that

$$\int_t^T dt' \bar{\mathbf{e}}(t') = \mathbf{e}(t) - \int_t^T dt' \mathbf{x}(t'). \tag{19}$$

Taking the transpose and insertion in equation 18 yields

$$\begin{aligned}\boldsymbol{\gamma}_{\boldsymbol{\theta}} &= -\int_0^T dt\, \left(\mathbf{e}^{\mathsf{T}}(t) - \int_t^T dt'\mathbf{x}^{\mathsf{T}}(t')\right) \mathbf{J}_1^{-1}(t)\left[\mathbf{K}(t) + \mathbf{J}_0(t)\mathbf{G}(t)\right] \\ &= -\int_0^T dt\, \mathbf{e}^{\mathsf{T}}(t)\mathbf{J}_1^{-1}(t)\mathbf{K}(t) - \int_0^T dt\, \mathbf{e}^{\mathsf{T}}\mathbf{J}_1^{-1}(t)\mathbf{J}_0(t)\mathbf{G}(t) \\ &\quad + \int_0^T dt \int_t^T dt'\mathbf{x}^{\mathsf{T}}(t')\mathbf{J}_1^{-1}(t)\left[\mathbf{K}(t) + \mathbf{J}_0(t)\mathbf{G}(t)\right]. \end{aligned} \tag{20}$$

Switching the order of integration in the final term yields

$$\begin{aligned} &\int_0^T dt \int_t^T dt'\mathbf{x}^{\mathsf{T}}(t')\mathbf{J}_1^{-1}(t)\left[\mathbf{K}(t) + \mathbf{J}_0(t)\mathbf{G}(t)\right] \\ &= \int_0^T dt\, \mathbf{x}^{\mathsf{T}}(t) \int_0^t dt'\mathbf{J}_1^{-1}(t')\left[\mathbf{K}(t') + \mathbf{J}_0(t')\mathbf{G}(t')\right] \\ &= \int_0^T dt\, \mathbf{x}^{\mathsf{T}}(t)\mathbf{G}(t), \end{aligned}$$

such that the last two terms in equation 20 disappear if

$$\mathbf{x}^{\mathsf{T}}(t) = \mathbf{e}^{\mathsf{T}}(t)\mathbf{J}_1^{-1}(t)\mathbf{J}_0(t). \tag{21}$$

This means that with this definition of $\mathbf{x}(t)$ the time integral of the gradient reduces to

$$\boldsymbol{\gamma}_{\boldsymbol{\theta}} = -\int_0^T dt\, \mathbf{e}^{\mathsf{T}}(t)\mathbf{J}_1^{-1}(t)\mathbf{K}(t), \tag{22}$$

in which the matrix $\mathbf{G}(t)$ no longer occurs.

5 Differential algebraic equations

In the case that $\mathbf{J}_1(t)$ is non-invertible, the previous analysis no longer holds. The system of equations is known as *differential algebraic equations* (DAE). This situation happens often in engineering applications such as finite-element methods, electric circuits etc. Often, it is possible to rewrite the equations as follows:

$$\begin{aligned} \dot{\mathbf{q}}(t) &= \mathbf{g}(\mathbf{q}(t), \mathbf{r}(t), \mathbf{s}(t), \boldsymbol{\theta}) \\ \mathbf{0} &= \mathbf{h}(\mathbf{q}(t), \mathbf{r}(t), \mathbf{s}(t), \boldsymbol{\theta}). \end{aligned} \tag{23}$$

Here, the bottom equation is known as the algebraic part of the system. It will impose restrictions on the variables $\mathbf{q}(t)$ through additional variables $\mathbf{r}(t)$. First of all we introduce the following notations:

$$\mathbf{G}_{\mathbf{q}}(t) = \frac{d\mathbf{q}(t)}{d\boldsymbol{\theta}}, \quad \mathbf{G}_{\mathbf{r}}(t) = \frac{d\mathbf{r}(t)}{d\boldsymbol{\theta}}, \tag{24}$$

$$\mathbf{K}^{\mathbf{g}}(t) = \frac{\partial \mathbf{g}(\mathbf{q}(t), \mathbf{r}(t), \mathbf{s}(t), \boldsymbol{\theta})}{\partial \boldsymbol{\theta}}, \quad \mathbf{K}^{\mathbf{h}}(t) = \frac{\partial \mathbf{h}(\mathbf{q}(t), \mathbf{r}(t), \mathbf{s}(t), \boldsymbol{\theta})}{\partial \boldsymbol{\theta}}, \tag{25}$$

$$\mathbf{J}^{\mathbf{g}}_{\mathbf{q}}(t) = \frac{\partial \mathbf{g}(\mathbf{q}(t), \mathbf{r}(t), \mathbf{s}(t), \boldsymbol{\theta})}{\partial \mathbf{q}(t)}, \quad \mathbf{J}^{\mathbf{g}}_{\mathbf{r}}(t) = \frac{\partial \mathbf{g}(\mathbf{q}(t), \mathbf{r}(t), \mathbf{s}(t), \boldsymbol{\theta})}{\partial \mathbf{r}(t)}, \tag{26}$$

$$\mathbf{J}^{\mathbf{h}}_{\mathbf{q}}(t) = \frac{\partial \mathbf{h}(\mathbf{q}(t), \mathbf{r}(t), \mathbf{s}(t), \boldsymbol{\theta})}{\partial \mathbf{a}(t)}, \quad \mathbf{J}^{\mathbf{h}}_{\mathbf{r}}(t) = \frac{\partial \mathbf{h}(\mathbf{q}(t), \mathbf{r}(t), \mathbf{s}(t), \boldsymbol{\theta})}{\partial \mathbf{r}(t)}. \tag{27}$$

Using these notations we can take the derivative of equation 23 w.r.t. $\boldsymbol{\theta}$:

$$\begin{aligned} \dot{\mathbf{G}}_{\mathbf{q}}(t) &= \mathbf{K}^{\mathbf{g}}(t) + \mathbf{J}^{\mathbf{g}}_{\mathbf{q}}(t)\mathbf{G}_{\mathbf{q}}(t) + \mathbf{J}^{\mathbf{g}}_{\mathbf{r}}(t)\mathbf{G}_{\mathbf{r}}(t) \\ \mathbf{0} &= \mathbf{K}^{\mathbf{h}}(t) + \mathbf{J}^{\mathbf{h}}_{\mathbf{q}}(t)\mathbf{G}_{\mathbf{q}}(t) + \mathbf{J}^{\mathbf{h}}_{\mathbf{r}}(t)\mathbf{G}_{\mathbf{r}}(t). \end{aligned} \tag{28}$$

This takes on the form of another set of equations. For the remainder of the derivation we will assume that $\mathbf{J}^{\mathbf{h}}_{\mathbf{r}}(t)$ is non-singular, which is true for DAEs of index 1. Now we can solve for $\mathbf{G}_{\mathbf{r}}(t)$ in the second equation, which yields

$$\mathbf{G}_{\mathbf{r}}(t) = -\left[\mathbf{J}^{\mathbf{h}}_{\mathbf{r}}(t)\right]^{-1}\left(\mathbf{K}^{\mathbf{h}}(t) + \mathbf{J}^{\mathbf{h}}_{\mathbf{q}}(t)\mathbf{G}_{\mathbf{q}}(t)\right), \tag{29}$$

leaving a single differential equation for $\mathbf{G}_{\mathbf{q}}(t)$. We wish to find out if we can redo the previous analysis in order to find a backpropagation algorithm. First we define output errors[2] on $\mathbf{q}(t)$ and $\mathbf{r}(t)$ as $\bar{\mathbf{e}}_{\mathbf{q}}(t)$ and $\bar{\mathbf{e}}_{\mathbf{r}}(t)$. The integral over the gradient is then given by

$$\boldsymbol{\gamma}_{\boldsymbol{\theta}} = \int_0^T dt \ \left(\bar{\mathbf{e}}^{\mathsf{T}}_{\mathbf{q}}(t)\mathbf{G}_{\mathbf{q}}(t) + \bar{\mathbf{e}}^{\mathsf{T}}_{\mathbf{r}}(t)\mathbf{G}_{\mathbf{r}}(t)\right) \tag{30}$$

Eliminating $\mathbf{G}_{\mathbf{r}}(t)$ leads to

$$\begin{aligned} \boldsymbol{\gamma}_{\boldsymbol{\theta}} &= -\int_0^T dt \bar{\mathbf{e}}^{\mathsf{T}}_{\mathbf{r}}(t)\left[\mathbf{J}^{\mathbf{h}}_{\mathbf{r}}(t)\right]^{-1}\mathbf{K}^{\mathbf{h}}(t) \\ &\quad + \int_0^T dt \left(\bar{\mathbf{e}}^{\mathsf{T}}_{\mathbf{q}}(t) + \bar{\mathbf{e}}^{\mathsf{T}}_{\mathbf{r}}(t)\left[\mathbf{J}^{\mathbf{h}}_{\mathbf{r}}(t)\right]^{-1}\mathbf{J}^{\mathbf{h}}_{\mathbf{q}}(t)\right)\mathbf{G}_{\mathbf{q}}(t). \end{aligned}$$

The first term no longer contains $\mathbf{G}_{\mathbf{q}}(t)$. In the second term we replace the expression between curved brackets by $\bar{\mathbf{e}}^{\mathsf{T}}_{x}(t)$. Changing $\mathbf{G}_{\mathbf{q}}(t)$ into its implicit integral solution and changing the order of integration then yields for the second term (ST):

$$\text{ST} = \int_0^T dt \int_0^T dt' \bar{\mathbf{e}}^{\mathsf{T}}_{x}(t')\left[\mathbf{K}^{\mathbf{g}}(t) + \mathbf{J}^{\mathbf{g}}_{\mathbf{q}}(t)\mathbf{G}_{q}(t) + \mathbf{J}^{\mathbf{g}}_{\mathbf{r}}(t)\mathbf{G}_{r}(t)\right] \tag{31}$$

We now assume that there exists a backpropagated error $\mathbf{e}_{\mathbf{q}}(t)$, defined by

$$\frac{d\mathbf{e}_{\mathbf{q}}(s)}{ds} = \bar{\mathbf{e}}_{x}(s) + \mathbf{x}(s), \tag{32}$$

such that we find that

$$\int_0^T dt' \bar{\mathbf{e}}_{x}(t') = \mathbf{e}_{\mathbf{q}}(t) - \int_0^T dt' \mathbf{x}(t'). \tag{33}$$

We can insert this expression into the second term and largely repeat the same derivation as before. The end result is given by:

$$\mathbf{x}^{\mathsf{T}}(t) = \mathbf{e}^{\mathsf{T}}_{\mathbf{q}}(t)\left(\mathbf{J}^{\mathbf{g}}_{\mathbf{q}}(t) - \mathbf{J}^{\mathbf{g}}_{\mathbf{r}}(t)\left[\mathbf{J}^{\mathbf{h}}_{\mathbf{r}}(t)\right]^{-1}\mathbf{J}^{\mathbf{h}}_{\mathbf{q}}(t)\right), \tag{34}$$

$$\boldsymbol{\gamma}_{\boldsymbol{\theta}} = \int_0^T dt \left(\mathbf{e}^{\mathsf{T}}_{\mathbf{q}}(t)\mathbf{K}^{\mathbf{g}}(t) - \left(\mathbf{e}^{\mathsf{T}}_{\mathbf{q}}(t)\mathbf{J}^{\mathbf{g}}_{\mathbf{r}}(t) - \bar{\mathbf{e}}^{\mathsf{T}}_{\mathbf{r}}(t)\right)\left[\mathbf{J}^{\mathbf{h}}_{\mathbf{r}}(t)\right]^{-1}\mathbf{K}^{\mathbf{h}}(t)\right). \tag{35}$$

[2]These are the gradients of a cost function $C(\mathbf{q}(t), \mathbf{r}(t), t)$ w.r.t. $\mathbf{q}(t)$ and $\mathbf{r}(t)$, respectively.

6 Delayed differential equations

Many real-world DSs are governed by interconnection delays. One important example is the finite speed of light, which matters greatly in high-speed electronics or photonics components. Delays are an interesting phenomenon in DSs as, at least in principle, they make the state space infinite-dimensional (the state history over the full length of the delay is now part of the "immediate" state of the DS). Furthermore, in contrast to discrete time DSs, in continuous time delays are differentiable, such that they too are parameters trainable by gradient descent. We define a set of N delays, denoted by $\mathbf{d} = [d_1, d_2, \cdots, d_N]$.
We now consider the following differential equation.

$$\dot{\mathbf{a}}(t) = \mathbf{f}(\mathbf{a}(t), \mathbf{s}(t), \mathbf{a_d}(t), \boldsymbol{\theta}). \tag{36}$$

Here, $\mathbf{a_d}(t)$ is a vertical concatenation of delayed versions of $\mathbf{a}(t)$:

$$\mathbf{a_d}(t) = [\mathbf{a}(t - d_1), \mathbf{a}(t - d_2), \cdots, \mathbf{a}(t - d_N)],$$

such that we assume that the evolution of $\mathbf{a}(t)$ depends on N delays. Note that, if the state evolution would happen to depend on delayed versions of the input signal, we can simply use all previous definitions, where we augment the input signal with the delayed versions. In this case, however, the situation is a little more complicated because we cannot reduce the equation to an ordinary differential equation, and we will need to redo the previous derivation. Before we begin we use the chain rule to find the total derivative of the state of the DS w.r.t. the parameters. We find that

$$\dot{\mathbf{G}}(t) = \mathbf{K}(t) + \mathbf{J}(t)\mathbf{G}(t) + \frac{\partial \mathbf{f}(\mathbf{a}(t), \mathbf{s}(t), \mathbf{a_d}(t), \boldsymbol{\theta})}{\partial \mathbf{a_d}(t)} \frac{d\mathbf{a_d}(t)}{d\boldsymbol{\theta}}. \tag{37}$$

We will use the following definitions:

$$\mathbf{J_d}(t) = \frac{\partial \mathbf{f}(\mathbf{a}(t), \mathbf{s}(t), \mathbf{a_d}(t), \boldsymbol{\theta})}{\partial \mathbf{a_d}(t)}, \tag{38}$$

and

$$\mathbf{G_d}(t) = \frac{d\mathbf{a_d}(t)}{d\boldsymbol{\theta}} = [\mathbf{G}(t - d_1), \mathbf{G}(t - d_2), \cdots, \mathbf{G}(t - d_N)]. \tag{39}$$

This reduces equation 37 to

$$\dot{\mathbf{G}}(t) = \mathbf{K}(t) + \mathbf{J}(t)\mathbf{G}(t) + \mathbf{J_d}(t)\mathbf{G_d}(t). \tag{40}$$

Consequently, changing $\mathbf{G}(t)$ by its implicit integral solution in equation 41 yields:

$$\boldsymbol{\gamma}_{\boldsymbol{\theta}} = \int_0^T dt\, \bar{\mathbf{e}}^{\mathsf{T}}(t) \int_0^t dt' \left(\mathbf{K}(t') + \mathbf{J}(t')\mathbf{G}(t') + \mathbf{J_d}(t')\mathbf{G_d}(t')\right), \tag{41}$$

We now wish to define a differential equation for the error backpropagation. We will for now assume it to be equal to

$$\frac{d\mathbf{e}(s)}{ds} = \bar{\mathbf{e}}(s) + \mathbf{x}(s), \tag{42}$$

where $\mathbf{x}(s)$, is the unknown part, again with $s = T - t$. We repeat the same derivation as before, but now exchange the integral over $\bar{\mathbf{e}}(t)$ by

$$\int_t^T dt' \bar{\mathbf{e}}^\mathsf{T}(t') = \mathbf{e}^\mathsf{T}(t) - \int_t^T dt' \mathbf{x}^\mathsf{T}(t'). \tag{43}$$

Changing the order of the integration, and substituting the integral over $\bar{\mathbf{e}}(t)$ finally yields:

$$\begin{aligned}
\boldsymbol{\gamma_\theta} &= \int_0^T dt \left(\mathbf{e}^\mathsf{T}(t) - \int_t^T dt' \mathbf{x}^\mathsf{T}(t') \right) [\mathbf{K}(t) + \mathbf{J}(t)\mathbf{G}(t) + \mathbf{J_d}(t)\mathbf{G_d}(t)] \\
&= \int_0^T dt\, \mathbf{e}^\mathsf{T}(t)\mathbf{K}(t) + \int_0^T dt\, \mathbf{e}^\mathsf{T}(t) [\mathbf{J}(t)\mathbf{G}(t) + \mathbf{J_d}(t)\mathbf{G_d}(t)] \\
&\quad - \int_0^T dt \int_t^T dt' \mathbf{x}^\mathsf{T}(t') [\mathbf{K}(t) + \mathbf{J}(t)\mathbf{G}(t) + \mathbf{J_d}(t)\mathbf{G_d}(t)],
\end{aligned}$$

and after switching the order of the integration in the final term and replacing the implicit integral solution of $\mathbf{G}(t)$, this becomes

$$\begin{aligned}
\boldsymbol{\gamma_\theta} &= \int_0^T dt\, \mathbf{e}^\mathsf{T}(t)\mathbf{K}(t) + \int_0^T dt\, \mathbf{e}^\mathsf{T}(t) [\mathbf{J}(t)\mathbf{G}(t) + \mathbf{J_d}(t)\mathbf{G_d}(t)] \\
&\quad - \int_0^T dt \mathbf{x}^\mathsf{T}(t)\mathbf{G}(t),
\end{aligned}$$

We again wish to obtain the following equality:

$$\boldsymbol{\gamma_\theta} = \int_0^T dt\, \mathbf{e}^\mathsf{T}(t)\mathbf{K}(t), \tag{44}$$

such that we require that

$$\int_0^T dt \mathbf{x}^\mathsf{T}(t)\mathbf{G}(t) = \int_0^T dt\, \mathbf{e}^\mathsf{T}(t) [\mathbf{J}(t)\mathbf{G}(t) + \mathbf{J_d}(t)\mathbf{G_d}(t)] \tag{45}$$

We start by making the following observation

$$\mathbf{J_d}(t)\mathbf{G_d}(t) = \sum_{i=1}^N \mathbf{J}_{d_i}(t)\mathbf{G}(t - \delta_i), \tag{46}$$

where

$$\mathbf{J}_{d_i}(t) = \frac{\partial \mathbf{f}(\mathbf{a}(t), \mathbf{s}(t), \mathbf{a_d}(t), \boldsymbol{\theta})}{\partial \mathbf{a}(t - d_i)}. \tag{47}$$

This means that we can rewrite the left hand side of equation 45 as

$$\begin{aligned}
\int_0^T dt \mathbf{x}^\mathsf{T}(t)\mathbf{G}(t) &= \int_0^T dt\, \mathbf{e}^\mathsf{T}(t) [\mathbf{J}(t)\mathbf{G}(t) + \mathbf{J_d}(t)\mathbf{G_d}(t)] \\
&= \int_0^T dt\, \mathbf{e}^\mathsf{T}(t) \left[\mathbf{J}(t)\mathbf{G}(t) + \sum_{i=1}^N \mathbf{J}_{d_i}(t)\mathbf{G}(t - d_i) \right].
\end{aligned}$$

Due to the fact that we have explicitly defined $\mathbf{G}(t)$ and $\mathbf{e}(t)$ to be equal to zero outside the range $0 < t < T$, we can integrate over each term in this equation separately and change the integration variable without changing the limits of integration, yielding:

$$\begin{aligned}\int_0^T dt\mathbf{x}^\mathsf{T}(t)\mathbf{G}(t) &= \int_0^T dt\ \mathbf{e}^\mathsf{T}(t)\mathbf{J}(t)\mathbf{G}(t) + \sum_{i=1}^N \int_0^T dt\ \mathbf{e}^\mathsf{T}(t)\mathbf{J}_{d_i}(t)\mathbf{G}(t-d_i) \\ &= \int_0^T dt\ \mathbf{e}^\mathsf{T}(t)\mathbf{J}(t)\mathbf{G}(t) + \sum_{i=1}^N \int_0^T dt\ \mathbf{e}^\mathsf{T}(t+d_i)\mathbf{J}_{d_i}(t+d_i)\mathbf{G}(t),\end{aligned}$$

which is fulfilled if

$$\mathbf{x}^\mathsf{T}(t) = \mathbf{e}^\mathsf{T}(t)\mathbf{J}(t) + \sum_{i=1}^N \mathbf{e}^\mathsf{T}(t+d_i)\mathbf{J}_{d_i}(t+d_i) \tag{48}$$

This result reduces the differential equation for $\mathbf{e}(s)$ to

$$\frac{d\mathbf{e}(s)}{ds} = \bar{\mathbf{e}}(s) + \mathbf{J}^\mathsf{T}(s)\mathbf{e}(s) + \sum_{i=1}^N \mathbf{J}_{d_i}^\mathsf{T}(s-d_i)\mathbf{e}(s-d_i), \tag{49}$$

such that it too is defined by a delayed differential equation.

Let us as an example consider what happens if we wish to find the gradient w.r.t. a certain delay value d_k. The only variable we need to consider is the part of $\mathbf{K}(t)$ which corresponds to the partial derivative of $\mathbf{f}$ w.r.t. d_k. As we assume that d_k is part of the parameter set, we also assume that $\mathbf{f}$ explicitly depends on d_k. We can write

$$\begin{aligned}\mathbf{K}_{d_k}(t) &= \frac{\partial \mathbf{f}(\mathbf{a}(t), \mathbf{s}(t), \mathbf{a_d}(t), \boldsymbol{\theta})}{\partial d_k} = \frac{\partial \mathbf{f}(\mathbf{a}(t), \mathbf{s}(t), \mathbf{a_d}(t), \boldsymbol{\theta})}{\partial \mathbf{a}(t-d_k)}\frac{\partial \mathbf{a}(t-d_k)}{\partial d_k} \\ &= -\mathbf{J}_{d_k}(t)\dot{\mathbf{a}}(t-d_k)\end{aligned}$$

7 Delayed differential algebraic equations

Obviously, certain DSs will have both an algebraic part and interconnection delays. We will limit ourselves here to one particular case which describes the photonic SOA networks.

We again use two variables $\mathbf{r}(t)$ and $\mathbf{q}(t)$, the first algebraic, the second dynamic. Furthermore we write $\mathbf{r_d}(t) = [\mathbf{r}(t-d_1), \mathbf{r}(t-d_2), \cdots, \mathbf{r}(t-d_N)]$. The DDAE we will consider is of the form

$$\begin{aligned}\dot{\mathbf{q}}(t) &= \mathbf{g}(\mathbf{q}(t), \mathbf{r_d}(t), \mathbf{s}(t), \boldsymbol{\theta}) \quad (50)\\ \mathbf{r}(t) &= \mathbf{h}(\mathbf{q}(t), \mathbf{r_d}(t), \mathbf{s}(t), \boldsymbol{\theta}). \quad (51)\end{aligned}$$

If external errors on $\mathbf{q}$ and $\mathbf{r}$ are written as $\bar{\mathbf{e}}_\mathbf{q}$ and $\bar{\mathbf{e}}_\mathbf{r}$, respectively, we can associate the backpropagated errors $\mathbf{e_q}$ and $\mathbf{e_r}$ as

$$\begin{aligned}\dot{\mathbf{e}}_\mathbf{q}^\mathsf{T}(t) &= \bar{\mathbf{e}}_\mathbf{q}^\mathsf{T} + \mathbf{e}_\mathbf{q}^\mathsf{T}(t)\mathbf{J}_\mathbf{q}^\mathbf{g}(t) + \mathbf{e}_\mathbf{r}^\mathsf{T}(t)\mathbf{J}_\mathbf{q}^\mathbf{h}(t) \quad (52)\\ \mathbf{e}_\mathbf{r}^\mathsf{T}(t) &= \bar{\mathbf{e}}_\mathbf{r}^\mathsf{T} + \sum_{i=1}^N \left(\mathbf{e}_\mathbf{q}^\mathsf{T}(t+d_i)\mathbf{J}_{d_i}^\mathbf{h}(t+d_i) + \mathbf{e}_\mathbf{r}^\mathsf{T}(t+d_i)\mathbf{J}_{d_i}^\mathbf{g}(t+d_i)\right), \quad (53)\end{aligned}$$

in which

$$\mathbf{J}^{\mathbf{g}}_{d_i}(t) = \frac{\partial \mathbf{g}(\mathbf{q}(t), \mathbf{r}_{\mathbf{d}}(t), \mathbf{s}(t), \boldsymbol{\theta})}{\partial \mathbf{r}(t-d_i)}, \qquad \mathbf{J}^{\mathbf{h}}_{d_i}(t) = \frac{\partial \mathbf{h}(\mathbf{q}(t), \mathbf{r}_{\mathbf{d}}(t), \mathbf{s}(t), \boldsymbol{\theta})}{\partial \mathbf{r}(t-d_i)}. \tag{54}$$

We end up with the following expression for the gradient

$$\boldsymbol{\gamma}_{\boldsymbol{\theta}} = \int_0^T dt \, \left(\mathbf{e}_{\mathbf{q}}^{\mathsf{T}}(t)\mathbf{K}^{\mathbf{g}}(t) + \mathbf{e}_{\mathbf{r}}^{\mathsf{T}}(t)\mathbf{K}^{\mathbf{h}}(t) \right)$$

The proof is lengthy but highly similar to the previous two derivations, therefore we omit it here. For the photonic networks, the dynamic variable $\mathbf{q}(t)$ corresponds to the free carrier densities of the SOAs, and the algebraic variable $\mathbf{r}(t)$ to the complex amplitudes of the incoming light (see main text for more details)
